# Multiplexed suspension array immunoassays for detection of antibodies to pneumococcal polysaccharide and conjugate vaccines

**DOI:** 10.3389/fcimb.2023.1296665

**Published:** 2023-11-15

**Authors:** Sherry A. Dunbar

**Affiliations:** Scientific Affairs, Luminex, A DiaSorin Company, Austin, TX, United States

**Keywords:** Streptococcus pneumoniae (pneumococcus), multiplex, serology, immunoassay, vaccine, Luminex, xMAP

## Abstract

Combination and polyvalent vaccines not only provide protection against several different pathogens at the same time but can also increase vaccine protection against pathogens that have closely related pathogenic strains or serotypes. Multiplexed serological testing is a preferred method for determining the efficacy of combination and polyvalent vaccines, as it reduces the need for conducting multiple individual assays to confirm immune responses and cross-reactivity, uses less sample, and can be faster, more reliable, and more cost-effective. Bead-based suspension array technologies, such as the Luminex^®^ xMAP^®^ Technology, are often used for development of multiplexed serological assays for various vaccine trials and for routine testing in clinical laboratories to determine immune status of vaccinated individuals. This article reviews publications describing the development and implementation of bead-based multiplexed serological assays for detection of immune responses to polyvalent polysaccharide and conjugate vaccines against *Streptococcus pneumoniae*. Many of these serological assays on the bead array platform have been further optimized and expanded over time and are still widely used today.

## Introduction

Combination and polyvalent vaccines not only provide protection against several different pathogens at the same time but can also increase vaccine protection against pathogens that have closely related pathogenic strains or serotypes. In particular, the use of polyvalent conjugate vaccines for *Streptococcus pneumoniae* is important due to the organism’s many serotypes, each with a distinct polysaccharide capsule (Hausdorff et al., 2000). In conjugate vaccines, the polysaccharide is coupled to a carrier protein which increases the immunogenicity and helps elicit a stronger response (Pichichero, 2013). However, serotype variability and evolution of new or altered serotypes makes it difficult to create a vaccine that can provide comprehensive and long-lasting coverage. Thus the polyvalent pneumococcal vaccines have also changed over time from the first 7-valent vaccine licensed in 2000, to the 14-valent vaccine, and the 23-valent vaccine licensed in 1983 (Daniels et al., 2016). With such broad coverage, testing the vaccine immune response to each serotype can be extremely time-consuming and laborious if each component must be assessed in an individual serological immunoassay. Therefore, multiplexed serological testing methods have been developed over the past two decades and are preferred for determining the efficacy of combination and polyvalent vaccines. Multiplexed immunoassays reduce the number of assays needed to confirm immune responses and cross-reactivity, use less serum, and can be performed faster. Testing in multiplex reduces the variables that must be controlled when performing individual tests and can thus be more reliable, and ultimately are more cost-effective than single-plex methods.

Bead-based suspension array technologies are often used for development of multiplexed serological assays to simultaneously assess immune responses to multiple antigens and have been used in vaccine trials, testing in clinical laboratories, epidemiological studies, and in basic immunological research. In particular, the xMAP^®^ microsphere technology from Luminex has been used extensively for multiplexed serological assays to detect immune responses to a variety of antigens, including pathogens, autoimmune markers, as well as human leukocyte antigen (HLA) and alloantigens, which is important for donor and recipient testing in transplantation (Das and Dunbar, 2020).

xMAP microspheres are polystyrene beads of the same size and with the same physical properties and surface composition but are internally dyed with two or three spectrally distinct fluorochromes (Graham et al., 2019). These internal classification dyes are excited at the same wavelength but have unique emission profiles that provide a unique “spectral address” for each individual microsphere region, or bead set, allowing each bead set to be differentiated from all others in the multiplexed reaction. Bead sets are covalently coupled with capture molecules specific to the target of interest and are then combined into a single multiplexed reaction. After binding the analytes present in a sample, a reporter fluorochrome is added to measure the binding that has occurred on the bead surface. Completed reactions are interrogated in a flow analysis instrument where the fluorescence of the internal dyes allows distinct analysis of the multiplex data, and the reporter fluorescence quantifies the result.

This article reviews selected publications describing the development and implementation of bead-based multiplexed serological assays for detection of immune responses to polyvalent vaccines against *Streptococcus pneumoniae* (**Table 1**). Many of the assays described have been further optimized and expanded over time or have been modified to have broad coverage for multiple vaccines used against different organisms, and many of these are still in use today. While initial studies focused on assay development and optimization, subsequent work has allowed implementation of these assays in clinical and research laboratories to characterize the immune responses to pneumococcal vaccines in various populations and to determine stability of immunity after vaccination and/or natural infection over time.

**Table 1:** Summary of the described literature on multiplexed pneumococcal serological assays.

Assay Development			
Chemistry/Features	Objectives	Outcome	Reference
14-plex, PnPS/PLL conjugate	Binding inhibition with homologous and heterologous serotypes	>95% homologous inhibition <15% heterologous inhibition	(Pickering et al., 2002)
25-plex, sodium periodate oxidation/ADH	Measure antibodies to 23-valent vaccine	LoDs ranged from 20-600 pg/mL	(Biagini et al., 2003)
9-plex, PnPS/PLL	Performance, comparison to ELISA	24-fold dilution linearity <30% heterologous inhibition 32.3-109.7 pg/ml LoD 5.2-8.34% intra-assay CV, 12.1-19.2% inter-assay CV	(Lal et al., 2005)
12-plex, DMTMM	Comparison to PLL, sodium periodate methods	Highest signals achieved with DMTMM	(Schlottmann et al., 2006)
19-plex, PnPS/PLL	Measure IgG and IgM to multiple bacterial subunit vaccines	3-log dynamic range ≥88% homologous inhibition for IgG >83% homologous inhibition for IgM	(Whitelegg et al., 2012)
Vaccine Research			
Vaccine Studied	Objectives	Outcome	Reference
23-valent	Feasibility of inhaled vaccine	Response to only a few serotypes, lower antibody levels	(Menzel et al., 2005)
7-valent	Response to booster vaccine	Protective antibody levels measured in most samples one month after booster	(Elberse et al., 2011)
7-valent	Response to 23-valent vaccine after priming with 7-valent vaccine	Combination of 7-valent and 23-valent vaccines did not improve responses	(Lazarus et al., 2011)
7-valent	Measure IgG and IgA in saliva and serum of children after vaccination	Significant increases in salivary antibodies IgG > IgA Saliva could be useful for surveillance	(Rodenburg et al., 2012)
7-valent	Determine if oligosaccharide supplementation improves antibody levels in preterm infants	Lower antibody levels in oligosaccharide-treated groups but similar to full-term and equal in preterm groups after booster	(van den Berg et al., 2015)
23-valent and 13-valent	Evaluate antibody response in Crohn’s disease patients with and without immunosuppressive treatment	Immunosuppressive treatment impaired antibody response to both vaccines Antibody further impaired by TNF-alpha treatment	(Kantsø et al., 2015)
13-valent	Evaluate immunogenicity in patients with end-stage renal disease on dialysis	Titers increased at 2 months post-vaccination but declined at 12 months	(Mitra et al., 2016)
7-valent and 23-valent	Determine persistence of vaccine response in sickle cell disease	Only 36% of patients had protective antibody levels at 37 months post-vaccination	(Santoro et al., 2016)
13-valent	Determine benefit of vaccination in pediatric oncology patients during or after immunosuppressive therapy	Single dose was safe and effective Ppatients should be vaccinated upon diagnosis and after completing immunosuppressive therapy	(Hung et al., 2017)
13-valent	Assay validation of bead-based assay to replace ELISA platform	LLoQ from 0.002-0.038 µg/ml >500-fold dynamic range <20% standard deviation	(Pavliakova et al., 2018)
13-valent	Bridging study for bead-based assay vs. ELISA	Linear, quantitative relationships for all serotypes Bead-based assay measured lower IgG levels in pre-immunization samples Recommended cutoffs from 0.10-0.35 µg/ml, depending on serotype	(Tan et al., 2018)
Epidemiology/Serosurveillance			
Vaccine and/or Assay	Objectives	Outcome	Reference
7- and 23-valent/9-plex	Evaluate IgG subclass and antibody responses in children with severe/recurrent bacterial infections	Responses to 7-valent serotypes increased IgG1 response observed for 7-valent conjugate vaccine IgG2 response observed for 23-valent PnPS vaccine and natural infection	(Uddin et al., 2006)
23-valent/14-plex	Measure post-vaccination antibody concentrations in patients highly susceptible to respiratory infections and with deficiencies in PnPS antibody response compared to healthy controls	11% of susceptible patients, 100% of antibody-deficient patients, and 4% of healthy controls lacked responses to 6 of 14 serotypes tested	(Borgers et al., 2010)
16-plex	Determine antibody levels in pregnant black, white, and Hispanic mothers and in cord blood	42% of all mothers with protective levels to all 16 serotypes Hispanic mothers more likely to have protective levels for 12 serotypes and less likely for 3 serotypes 33% of infants had protective antibody levels	(Choudhury et al., 2012)
14-plex	Determine proportion of CAP due to pneumococcal infection	Significant response detected in 45% of pneumococcal pneumonia patients, in 5% of non-pneumococcal pneumonia, and in 28% with unidentified cause of CAP	(van Mens et al., 2011)
13-valent	Determine vaccine effectiveness in preventing CAP in an adult cohort in Italy	Effectiveness was 33.2% for any serotype and 38.1% for vaccine serotypes Effectiveness was 42.3% in age groups with higher vaccine uptake Effectiveness was 88.1% for community-managed cases and 91.7% after control for comorbidities	(Prato et al., 2018)
Research Studies			
Vaccine and/or Assay	Objectives	Outcome	Reference
12-plex	Compare IgG, IgA, and IgM levels in saliva vs. serum	Levels in saliva correlated with serum and was highest for IgA Salivary IgG and IgA could distinguish protective levels	(Heaney et al., 2018)
23-valent/12-plex	Determine if acute, moderate exercise prior to vaccination could enhance antibody response	IgM and IgG were increased in response to vaccine No difference between exercise and no exercise groups	(Long et al., 2012)
23-valent/12-plex	Determine if exercise intervention could enhance response to vaccination in middle-aged, sedentary women	Exercise intervention improved subjective and objective physical and psychological factors but did not affect antibody response	(Long et al., 2013)
23-valent/12-plex	Determine the benefit of acute exercise immediately before vaccination	Across all subjects, exercise groups showed greater antibody responses than control groups	(Edwards et al., 2012)
23-valent/14-plex	Determine if antibody levels could predict outcome in kidney transplant patients	Pre-vaccination antibodies were higher in transplant patients with poorer survival, but of no predictive value post-vaccination	(Lindemann et al., 2013)

## Multiplexed pneumococcal serological assay development

The first multiplexed microsphere-based immunoassay for measuring IgG antibodies to *S. pneumoniae* was described in 2002 by Pickering et al. (2002). The investigators developed a multiplexed indirect immunoassay to simultaneously measure serum IgG to 14 pneumococcal polysaccharides (PnPS). The PnPS were covalently bound to poly-L-lysine (PLL) using cyanuric chloride as the coupling agent. Each PnPS/PLL conjugate was coupled to a unique color-coded microsphere set using a two-step 1-ethyl-3-(3-dimethylaminopropyl)-carbodiimide hydrochloride (EDC) and sulfo-N-hydroxysulfosuccinimide (sulfo-NHS) procedure (Fulton et al., 1997). Binding inhibition studies were performed using serum samples from adult vaccinated individuals. With the exception of serotypes 9V and 9N (which are closely related), they achieved >95% inhibition by homologous serotypes. Less than 15% inhibition was found with heterologous serotypes for all 14 serotypes. Cross-reacting antibodies caused ≥50% heterologous inhibition with some serotypes and could not be removed by preabsorption with pneumococcal C-polysaccharide. However, they could be removed by an additional preabsorption with serotype 22F PnPS. The multiplexed assay showed good overall agreement with the enzyme-linked immunosorbent assay (ELISA) that was recommended at the time for evaluating pneumococcal vaccine immunogenicity. Subsequently, Pickering and colleagues described false positive reactivity that could occur due to nonspecific binding to the microspheres or reactivity to bovine serum albumin (BSA) present in the bead storage buffer (Pickering et al., 2010). They found that the nonspecific reactivity could be eliminated by replacing the BSA with BSA-free StabliGuard immunoassay stabilizer (SurModics, Inc., Eden Prairie, MN) and might be applicable to other bead-based serological assays.

In 2003 Biagini et al., developed a 25-plex PnPS assay for simultaneous measurement of antibodies to the 23 serotypes included in the PNEUMOVAX 23 vaccine (Merck & Co., Inc., North Wales, PA) (Biagini et al., 2003). This assay also included an internal control to evaluate pneumococcal cell wall polysaccharide preadsorption and a control using serotype 25 (not included in the vaccine), to assess inter-assay reproducibility. For conjugation, PnPS were first oxidized with sodium periodate to generate reactive aldehyde groups suitable for coupling to molecules that contain amine or hydrazide (Hermanson, 2013). The carboxylated microspheres were then modified by coupling adipic acid dihydrazide (ADH) using 1-ethyl-3-(3-dimethylaminopropyl)-carbodiimide hydrochloride (EDC) to provide a 10-atom spacer with an active amine group for subsequent covalent coupling to the oxidized PnPS. The assay was standardized using a reference anti-pneumococcal serotype standard. The investigators found that the limits of detection (LoD) ranged from 20 pg/ml for PnPS 3 to 600 pg/ml for PnPS 14. The authors concluded that compared to standard ELISAs, the method had several advantages for measuring anti-PnPS IgG levels, such as faster time-to-result, less sample volume required, and an equal or better sensitivity with an increased dynamic range.

Lal and colleagues developed a multiplexed serological assay for simultaneous quantitation of IgG to nine pneumococcal serotypes (1, 4, 5, 6B, 9V, 14, 18C, 19F, 23F) (Lal et al., 2005). Pneumococcal polysaccharides were conjugated to poly-L-lysine (PLL) and covalently attached to fluorescent microspheres as previously described (Pickering et al., 2002). The coupled beads were assessed using 89-SF standard serum. The multiplexed assay was linear over a 24-fold range of serum dilution and comparison of the individual single-plex assays to the nine-plex assay demonstrated no interference between bead sets. The assay was specific with <30% heterologous inhibition, highly sensitive with LoDs from 32.3 to 109.7 pg/ml, and very reproducible with intra-assay coefficient of variation (CV) 5.2% to 8.34% and inter-assay CV at 12.1% to 19.2%. Results for clinical samples showed good correlation between the multiplexed assay and single-plex ELISAs. Correlation coefficients (*R*) were 0.91 to 0.96 and the slopes ranged from 0.82 to 1.07. The authors also added four additional, previously developed bead sets coupled with meningococcal polysaccharides to the PnPS multiplex and found no interference with the detection of pneumococcal or meningococcal IgG levels.

Schlottmann and coworkers reported a novel chemistry for conjugating PnPS to carboxylated microspheres (Schlottmann et al., 2006). In this method, a set of 12 PnPS were modified with 4-(4,6-dimethoxy (Hausdorff et al., 2000; Daniels et al., 2016; Graham et al., 2019)triazin-2-yl)-4-methyl-morpholinium (DMTMM) for subsequent conjugation to the microspheres. Several conjugation methods were compared: PLL/cyanuric chloride (Pickering et al., 2002), sodium periodate oxidation (Biagini et al., 2003), passive adsorption to carboxylated microspheres, DMTMM conjugation to carboxylated microspheres, and DMTMM conjugation to microspheres that were amino-modified with adipic dihydrazide (ADH). The authors evaluated each of these methods for robustness, reproducibility, and the effect on PnPS antigenicity in a multiplexed assay format. While they found that the PLL method had better overall coupling efficiency and better specificity compared to the oxidation method, it was not reproducible across different coupling episodes. PnPS did bind to the microspheres through passive adsorption, but the signals were less than those obtained with DMTMM, and the signals obtained for DMTMM with amino-modified microspheres was less than that with the carboxylated microspheres. When compared to PLL, the DMTMM method using carboxylated microspheres achieved the highest signals and the results were reproducible across multiple operators. Analytical sensitivities ranged from 0.6 to 53.2 ng/ml, depending on the particular PnPS, and the assay was quantitative across a 3.5 to 4 log dynamic range. Additionally, the method did not appear to affect antigenicity and was specific with ≥95% homologous inhibition for all PnPS types and 15-20% heterologous inhibition in only three instances.

Whitelegg et al. subsequently developed a multiplexed bead-based assay for measuring IgG and IgM antibodies to 19 antigens contained in groups of bacterial subunit vaccines including pneumococcal, meningococcal, and *Haemophilus* polysaccharides, and tetanus and diphtheria toxoids (Whitelegg et al., 2012). Polysaccharides were conjugated using the PLL method (Pickering et al., 2002; Lal et al., 2005) and the assay was used to measure specific IgG and IgM antibody levels in serum from 193 healthy adult donors. IgG and pneumococcal IgM concentrations could be measured across a 3-log dynamic range that included the protective threshold of IgG levels for each antigen. Comparison to the single-plex assays showed little interference in antibody measurements in the multiplexed assay. Specificity was determined by preincubating control serum with each of the 19 antigens before performing the assay. There was no cross-reactive IgG detected and the signal was reduced to ≤12% of the unadsorbed level for homologous antigen but the majority remained at >60% when adsorbed with the heterologous antigen. Cross-reactive IgM was found for three of the antigens, but signal was reduced to <17% for all others when preadsorbed with homologous antigen. They concluded that the assay was suitable for adoption by clinical laboratories to assess immune response to vaccinations and measure absolute antibody levels to these antigens.

## Vaccine research using multiplexed pneumococcal polysaccharide assays

These well-characterized assays have been deployed in numerous studies to explore the safety and immunogenicity of PnPS vaccines in a variety of patient populations and vaccination protocols. In 2005, Menzel and colleagues used a 9-plex bead-based PnPS assay to evaluate the feasibility of PnPS vaccination by inhalation (Menzel et al., 2005). Patients were randomized to compare alveolar vaccination, bronchial vaccination, and the standard intramuscular vaccination. They used an IgG enzyme immunoassay (EIA) to measure total pneumococcal-specific IgG and selected four responders from each cohort to determine serotype-specific IgG using the multiplexed bead array. The data showed that responses were restricted to a few serotypes in the inhalation groups (1 to 4 of 9 serotypes) and was broader for the intramuscular group (6 to 9 of 9 serotypes). Overall, the authors found that the vaccine could be administered safely by controlled inhalation but induced lower serum antibody responses as compared to intramuscular vaccination.

Elberse et al. used an optimized 13-plex PnPS assay to study the response to booster vaccination following immunization to the seven-valent PnPS vaccine (Elberse et al., 2011). Antibody concentrations were measured in 188 serum samples obtained pre- and post-booster vaccination at 11 months after a primary series of the vaccine. The results of the bead-based assay were compared with those of the ELISA for the serotypes included in the vaccine and for a non-vaccine serotype (6A). The concentrations of the antibodies as determined by the bead-based assay were slightly higher than those determined by ELISA but the correlations between the assays were good, with R2 values ranging from 0.84 to 0.91 for all serotypes except 19F (R2 = 0.70). Most of the serum samples had antibody concentrations above the protective concentration for the vaccine serotypes one month after booster. However, based on the differences in the concentrations determined by ELISA and the bead-based assay, the authors suggested a new protective cutoff concentration may need to be determined and may need to be adjusted for each serotype.

Lazarus and coworkers used the assay described by Lal (Lal et al., 2005) in a randomized study of adults 50-70 years of age to determine if priming with the 7-valent pneumococcal conjugate vaccine (PCV7) could improve the immunogenicity of 23-valent polysaccharide vaccine (23vP) for PCV7 serotypes, and to investigate whether PCV7 could be used to reverse reduced responsiveness to 23vP (Lazarus et al., 2011). The investigators conducted a randomized study that compared three vaccine schedules, consisting of two doses of PCV7 and one dose of 23vP administered over one year. Blood samples were obtained before and one month after each vaccination. The results showed that vaccination with 23vP after priming with two doses of PCV7 produced significantly higher antibody concentrations for three of the PCV7 serotypes as compared to vaccination with one dose of 23vP. The same immunogenicity was also achieved with a single dose of PCV7. Previous vaccination with 23vP weakened the antibody response to subsequent PCV7 vaccination and could not be restored by additional doses of PCV7. The authors concluded that vaccination schedules combining PCV7 and 23vP do not improve immune responses in adults compared to a single dose of 23vP for most of the PCV7 serotypes.

Rodenburg et al. used the multiplexed PnPS assay described by Pickering (Pickering et al., 2002) to measure anti-pneumococcal IgG and IgA in saliva and serum of children who received 2 doses (2 and 4 months of age) or 3 doses (2, 4, and 11 months of age) of PCV7 (Rodenburg et al., 2012). Paired saliva samples were collected from 188 children at 12 and 24 months of age and serum samples were also collected from 15 children. At 12 months, both vaccine groups had higher levels of IgG against vaccine serotypes in serum and saliva as compared with unvaccinated controls. Antibody levels were sustained until 24 months for most serotypes and although salivary IgG was 10- to 20-fold lower than in serum, the serum and saliva levels were highly correlated. Serum and salivary IgA levels were higher in both vaccine groups at 12 months with the exception of serotype 19F. Higher salivary IgA levels remained for 24 months for most serotypes in the 3-dose group, but not in the 2-dose group. PCV7 vaccination resulted in significant increases in pneumococcal-specific salivary IgG and IgA but were more prominent for IgG compared to the control group. Since the salivary antibody levels correlated well serum IgG, the authors suggest that saliva testing could also prove useful for surveillance of pneumococcal antibody levels.

van den Berg and colleagues used the multiplexed PnPS assay described by Elberse (Elberse et al., 2011) to determine the effect of oligosaccharide supplementation on antibody levels in preterm infants (<32 weeks gestational age or birth weight <1500 g) after vaccination with the Prevenar-7^®^ pneumococcal conjugate vaccine from Pfizer, Inc. (Pearl River, New York) (van den Berg et al., 2015). Following three primary vaccinations in 113 preterm infants, the oligosaccharide-treated groups had lower antibody levels than the placebo group at 5 months, but these levels were similar to those in a full-term control group. After receiving a booster vaccination at 11 months, antibody levels were the same between the preterm oligosaccharide-treated and the preterm placebo group. The authors suggested that lower IgG levels to vaccine serotypes in the oligosaccharide supplemented infants at 5 months might reflect either a direct or indirect (through alteration of microbiota) immunomodulation by neutral and acidic oligosaccharides in the first months of life. In addition, they observed that the transplacental transport of pneumococcal antibodies was lower in preterm infants than in full term infants.

These assays have also been used in many studies to evaluate immune responses to PnPS vaccines in a variety of different patient populations with conditions that can impair the immune response, such as autoimmune diseases, cancer, and transplantation. Kantsø et al. evaluated specific antibody response to two PnPS vaccines (23- and 13-valent) in Crohn’s disease patients with and without immunosuppressive treatment four weeks after vaccination (Kantsø et al., 2015). They used a laboratory-developed bead-based serological assay following the PnPS/PLL method (Pickering et al., 2002; Lal et al., 2005). Comparison between treatment groups showed that immunosuppressive treatment impaired the antibody response to both vaccines and that TNF-alpha treatment further impaired the response as compared to untreated patients.

Mitra and colleagues evaluated the immunogenicity of the 13-valent pneumococcal conjugate vaccine in patients with end-stage renal disease who were on dialysis, as these patients are at risk of pneumococcal disease (Mitra et al., 2016). Patients were given a single dose of vaccine and the serum antibody concentrations were measured using the multiplexed bead-based platform at baseline, and at 2 and 12 months after vaccination. Increased concentrations of antibodies to the vaccine serotypes were demonstrated at 2 months post-vaccination but declined by 38 to 72% at 12 months. They proposed further study to understand the reduced titers after 12 months.

Santoro et al. conducted a study to determine persistence of vaccine responses in patients with sickle cell disease (Santoro et al., 2016). The study assessed the vaccine response in sickle cell patients who were vaccinated at 2 and 5 years of age and then every 5 years. Anti-pneumococcal antibody titers were measured using the multiplexed bead assay described by Pickering (Pickering et al., 2002). The results showed a significant percentage of patients did not maintain a sufficient vaccine response for 5 years. Only 36% had protective levels of anti-pneumococcal antibody at 37 months post-vaccination and of these, 64% demonstrated a vaccine response to fewer than 25% of the tested serotypes. The authors recommended further investigation to determine an optimal vaccine schedule and to monitor anti-pneumococcal antibody titers in patients who are at risk for pneumococcal disease.

Hung and colleagues conducted a prospective study in pediatric oncology patients to determine the benefit of pneumococcal vaccination in patients receiving immunosuppressive therapy or who were within one year of completing therapy (Hung et al., 2017). IgG titers to 12 PnPS serotypes were measured in blood samples taken before and 4 weeks after vaccination using the bead-based multiplex platform. Prior to vaccination, ≤50% of the 82 patients had protective antibody titers for 8 serotypes in the completed treatment group (36 patients) and 10 serotypes in the active treatment group (46 patients) before vaccination. Post-vaccination, ≥70% had protective antibody titers for 9 and 11 serotypes in the active and completed groups, respectively. They concluded that a single dose of the 13-valent pneumococcal vaccine was safe and effective in pediatric oncology patients, and they should receive the vaccine upon cancer diagnosis and then again after completing immunosuppressive therapy.

In 2018, Pavliakova and colleagues at Pfizer reported the validation of a multiplexed direct bead-based immunoassay to replace the standard pneumococcal ELISA platform (Pavliakova et al., 2018). The multiplexed assay simultaneously measures the concentration of serum IgG antibodies specific for pneumococcal capsular polysaccharide serotypes 1, 3, 4, 5, 6A, 6B, 7F, 9V, 14, 18C, 19A, 19F, and 23F. This assay was developed using the PnPS/PLL method (Pickering et al., 2002). Assay validation was performed using residual human serum samples obtained from vaccine clinical studies. The lower limit of quantitation (LLoQ) for all serotypes covered in the 13-plex assay ranged from 0.002 to 0.038 µg/ml serum IgG. The dynamic range of the assay was >500-fold and the variability was <20% relative standard deviation for all serotypes. The assay is capable of generating up to 143 test results in a single 96-well plate and the authors concluded it was a suitable replacement for ELISA in evaluating vaccine clinical trials.

Tan et al. described the results of a study to bridge the World Health Organization (WHO) pneumococcal ELISA platform to the validated bead-based 13-plex direct immunoassay platform developed by Pfizer (Tan et al., 2018). Serum samples were selected from four pneumococcal vaccine clinical trials on the basis of serotype-specific IgG concentrations determined by ELISA. A comparison of assay results from the two platforms on 1,528 samples showed clear and robust linear quantitative relationships across all 13 serotypes. Lower IgG antibody concentrations in pre-immunization samples were measured in the bead-based assay and allowed better differentiation between pre-immunization and post-immunization samples with low titers. The results showed that the established protective threshold concentration of 0.35 µg/ml of serotype-specific serum IgG antibodies was appropriate for the multiplexed assay for 10 of the serotypes; however they recommended using the assay cutoff values of 0.23, 0.10, and 0.12 µg/ml for 3 serotypes.

## Epidemiology and serosurveillance using multiplexed pneumococcal polysaccharide assays

Uddin and collaborators evaluated the IgG subclass and pneumococcal serotype-specific antibody responses in vaccinated children with history of severe or recurrent bacterial infections (Uddin et al., 2006). Subjects were vaccinated with two doses of the PCV7 conjugate vaccine and one dose of the 23vP vaccine. Using the 9-plex assay described Lal (Lal et al., 2005), the investigators found that the responses to the serotypes in PCV7 increased significantly post-vaccination but responses to two serotypes only included in 23vP were low. ELISA assays showed that children mounted an IgG1 antibody response after immunization with the PCV7 conjugate vaccine, whereas IgG2 responses are commonly found with PnPS vaccines and in natural infection with *S. pneumoniae*. IgG1 is the dominant antibody produced in response to vaccines containing protein antigens, such as conjugate vaccines, and is associated with strong, specific responses that can provide targeted and long-lasting immunity whereas IgG2 antibodies are more common in response to carbohydrate antigens (Vidarsson et al., 2014).

Borgers et al. used a multiplexed bead-based serological assay to measure post-vaccination antibody concentrations in healthy adults and children, patients with high susceptibility to respiratory infection, and patients with deficient anti-PnPS antibody response (Borgers et al., 2010). The cutoff values for serotype-specific antibody levels were determined in healthy controls. Of the susceptible group, 11% failed to respond to at least 6 of 14 serotypes tested, compared to 4% of the controls. In the antibody-deficient group, 100% failed to respond to at least 6 of the 14. They concluded that the multiplexed assay can be used to identify individuals with low responses to unconjugated pneumococcal vaccines.

Choudhury et al. used a 16-plex bead-based assay to determine *S. pneumoniae* antibody levels in pregnant non-Hispanic black, non-Hispanic white, and Hispanic mothers, and in cord blood specimens (Choudhury et al., 2012). For all mothers, 42% had protective antibody levels to all 16 serotypes. Hispanic mothers were more likely than non-Hispanic mothers to have protective antibody levels for 12 of the serotypes, but less likely to have protective levels for 3 of the serotypes (9V, 12F, and 18C). Cord blood analysis revealed that 33% of infants had protective antibody levels. Hispanic infants had higher occurrence of protective levels to all serotypes except 11A, 14, 18C, and 23F, while non-Hispanic black infants had higher prevalence of protective immunity to 11A, 14, and 18C, and non-Hispanic white infants only had a higher protective level to serotype 23F.

A longitudinal analysis of anti-pneumococcal antibodies during community-acquired pneumonia (CAP) was conducted by van Mens and colleagues to determine the proportion of CAP due to pneumococcal infections (van Mens et al., 2011). A 14-plex bead-based assay was used to measure pneumococcal antibodies in the serum of hospitalized CAP patients. A significant pneumococcal immune response was defined as a ≥2-fold increase in antibody concentrations against a single serotype between day 1 and day 30 and a final concentration above >0.35 µg/ml. A significant immune response was detected in 45% of pneumococcal pneumonia patients, in 5% of non-pneumococcal pneumonia patients, and in 28% of patients where the causative agent for CAP was not identified. The study showed that a substantial portion of pneumococcal pneumonia patients do not elicit a serotype-specific immune response.

Prato et al. conducted a case-control study in a 2-year prospective cohort in Italy to determine effectiveness of the 13-valent vaccine in preventing CAP in adults (Prato et al., 2018). The overall vaccine effectiveness was 33.2%, irrespective of serotype, and 38.1% for vaccine serotypes. The effectiveness was higher at 42.3% for the vaccine serotypes in age groups with higher vaccine uptake. For a subgroup of cases that were managed in the community, the overall vaccine effectiveness due to any pneumococcal strain was 88.1% and 91.7% when they controlled for underlying comorbidities. The results obtained here were not statistically significant however, the authors recommend larger studies to verify the direct benefits of vaccination.

## Research studies using multiplexed pneumococcal polysaccharide assays

The multiplexed bead-based PnPS assay platform has also proven useful for measuring serotype-specific antibodies in a variety of clinical research studies. Heaney and coworkers conducted a study to determine if anti-pneumococcal antibody levels in saliva mirrored concentrations in serum (Heaney et al., 2018). They used the 12-plex assay described by Whitelegg (Whitelegg et al., 2012) to measure IgG, IgA and IgM levels in paired saliva and serum samples in 72 healthy adults. They found that the antibody levels in saliva were positively correlated with serum across immunoglobulin classes and was strongest for IgA. Individuals with protective serum antibody levels showed significantly higher IgG and IgA in saliva. Salivary IgG and IgA antibodies were able to distinguish between those with and without protective levels in serum for most serotypes. The findings suggested that anti-pneumococcal IgG and IgA in saliva may be suitable surrogate markers for antibody in serum.

Long et al. examined whether an acute moderate intensity aerobic exercise (walking) prior to vaccination could enhance pneumococcal antibody response (Long et al., 2012). Sixty young adults and 60 older adults participated in the study. A 12-plex assay using the PnPS/PLL chemistry (Pickering et al., 2002) was used to measure serotype-specific antibody levels prior to 45 min of exercise intervention and in resting controls. After exercise, all participants received a full-dose pneumococcal vaccination and a half-dose influenza vaccination. Antibody titers were determined again after four weeks. Both IgM and IgG antibody titers were increased to the pneumococcal vaccine. However, there was no significant difference between the groups. The authors suggest that higher intensity exercise would be needed to enhance antibody response to vaccination.

A subsequent study reported by the same group assessed whether exercise intervention could enhance the antibody response to pneumococcal vaccination in middle-aged, sedentary women (Long et al., 2013). Eighty-nine subjects participated in the study where 44 completed a 16-week walking exercise program and 45 served as controls. The 23vP vaccine was administered at 12 weeks and antibody levels were determined pre-vaccination and at 1- and 6-months post-vaccination. Physical and psychological factors were measured before and after intervention and participants in the exercise group had better subjective and objective physical activity levels and reported a better quality of life. However, no significant effects on antibody response to pneumococcal vaccination demonstrated in this study.

Edwards and collaborators conducted a similar study to determine the benefit of acute exercise immediately prior to vaccination to enhance the immune response (Edwards et al., 2012). They evaluated the effect of exercise on response to either a full- or half-dose of the 23vP vaccine in 133 young, healthy adults. Subjects were randomized according to exercise or control groups receiving full- or half-dose vaccination. Exercise groups completed a 15 min arm and shoulder exercise routine while control groups rested quietly. Antibody levels to pneumococcal serotypes were determined at baseline and one month using the 12-plex bead-based assay described by Whitelegg (Whitelegg et al., 2012). Across all subjects, the exercise groups showed greater antibody responses than the control groups and significantly higher responses for serotypes 1, 3, 4, and 9V. The group-by-time interaction effect was significant in the half-dose group, confirming the greater responses observed in the exercise group, but the main effect was similar for the full-dose group. The investigators concluded that the data indicate that exercise is useful as a vaccine adjuvant, particularly in weaker responses, and the potential benefit to improve protective immunity indicates acute exercise prior to vaccination should be evaluated in at-risk populations.

Lindemann et al. reported a study using bead-based multiplexed serological tests to determine if anti-pneumococcal and anti-HLA antibody levels could predict outcome in kidney transplant patients (Lindemann et al., 2013). Blood was collected from 49 patients pre-vaccination and at 1- and 15-months post-vaccination. Kidney function was unchanged at month 1 post-vaccination compared to pre-vaccination, and no rejection was observed within 5 years after vaccination. The median time between the transplant and vaccination was 6.5 years and the follow-up post-vaccination was 13 years. The data showed the pre-vaccination antibodies against pneumococci were significantly higher in transplant recipients with poorer patient survival but at months 1 and 15 post-vaccination, pneumococcal antibodies were of no predictive value, suggesting that only naturally acquired antibodies could be important for assessing outcomes. However, Class I HLA antibodies were significantly associated with patient survival both prior to and at 15 months post-vaccination.

## Conclusion

Since the first report in 2002 of the development of a multiplexed bead-based serological assay for PnPS-specific antibody detection, more than 80 articles have been published describing use of this method for the study of vaccine immune responses to multi-valent polysaccharide and conjugate pneumococcal vaccines. The literature highlights the advances in technology that now allow multiplexed analysis of immune responses to multiple pneumococcal serotypes simultaneously. This change in methodology also triggered some challenges to be overcome in the transition away from the traditional single-plex ELISA to the multiplexed format. Antibody concentrations measured by the bead-based assay tend to be higher than that measured by ELISA, suggesting that new serotype-specific cutoffs for protective levels were needed (Elberse et al., 2011). While a single cutoff may be suitable for many serotypes, some serotypes may require different cutoffs (Tan et al., 2018). It would in fact be extremely challenging to assign a single universal protective threshold, as the predominant serotypes evolve and change over time and therefore the cutoffs need to change as new data become available to inform vaccine strategy.

The Luminex xMAP bead-based suspension array platform is considered a benchmark for multiplexed serological testing for many infectious diseases, autoimmune disorders, and in transplantation for screening donors and monitoring recipients (Das and Dunbar, 2020). This paper reviews selected studies on multiplexed bead based PnPS serological assays and includes publications describing the history of the assay development, optimization, and validation, and how these assays have been used in vaccine studies and to evaluate pneumococcal vaccine responses in various patient populations. These assays are also used for epidemiological studies and for conducting serosurveillance of pneumococcal infections in various settings. Finally, studies showing how these assays have been deployed in biological and clinical immunology research are also featured. The large body of published literature on these multiplexed bead array assays proves the importance of this technology to support the understanding of the safety, immunogenicity, and efficacy of vaccines against *S. pneumoniae*.

## Author contributions

SD: Writing – original draft.
